# Genome analysis of *Flaviramulus ichthyoenteri* Th78^T^ in the family *Flavobacteriaceae*: insights into its quorum quenching property and potential roles in fish intestine

**DOI:** 10.1186/s12864-015-1275-0

**Published:** 2015-02-05

**Authors:** Yunhui Zhang, Jiwen Liu, Kaihao Tang, Min Yu, Tom Coenye, Xiao-Hua Zhang

**Affiliations:** College of Marine Life Sciences, Ocean University of China, 5 Yushan Road, Qingdao, 266003 P.R. China; Laboratory of Pharmaceutical Microbiology, Ghent University, 9000 Gent, Belgium

**Keywords:** *Flaviramulus ichthyoenteri*, Genome analysis, Quorum quenching, Intestine microbes

## Abstract

**Background:**

Intestinal microbes play significant roles in fish and can be possibly used as probiotics in aquaculture. In our previous study, *Flaviramulus ichthyoenteri* Th78^T^, a novel species in the family *Flavobacteriaceae*, was isolated from fish intestine and showed strong quorum quenching (QQ) ability. To identify the QQ enzymes in Th78^T^ and explore the potential roles of Th78^T^ in fish intestine, we sequenced the genome of Th78^T^ and performed extensive genomic analysis.

**Results:**

An *N*-acyl homoserine lactonase FiaL belonging to the metallo-β-lactamase superfamily was identified and the QQ activity of heterologously expressed FiaL was confirmed *in vitro*. FiaL has relatively little similarity to the known lactonases (25.2 ~ 27.9% identity in amino acid sequence). Various digestive enzymes including alginate lyases and lipases can be produced by Th78^T^, and enzymes essential for production of B vitamins such as biotin, riboflavin and folate are predicted. Genes encoding sialic acid lyases, sialidases, sulfatases and fucosidases, which contribute to utilization of mucus, are present in the genome. In addition, genes related to response to different stresses and gliding motility were also identified. Comparative genome analysis shows that Th78^T^ has more specific genes involved in carbohydrate transport and metabolism compared to other two isolates in *Flavobacteriaceae*, both isolated from sediments.

**Conclusions:**

The genome of Th78^T^ exhibits evident advantages for this bacterium to survive in the fish intestine, including production of QQ enzyme, utilization of various nutrients available in the intestine as well as the ability to produce digestive enzymes and vitamins, which also provides an application prospect of Th78^T^ to be used as a probiotic in aquaculture.

**Electronic supplementary material:**

The online version of this article (doi:10.1186/s12864-015-1275-0) contains supplementary material, which is available to authorized users.

## Background

The family *Flavobacteriaceae*, a major phylogenetic lineage in the phylum *Bacteroidetes* [1,2], currently consists of over 110 genera (http://www.bacterio.cict.fr) comprising diverse bacteria which occur primarily in various marine environments from surface water to deep-sea sediment [3], as well as other temperate and polar habitats in terrestrial and freshwater ecosystems. Members of the *Flavobacteriaceae* are well-known for their capacity of degrading many different polysaccharides, proteins and other biopolymers [4,5]. Because of their roles as decomposers in marine environment, they have attracted considerable interest in recent years [6-9]. Whole genome analyses of some species of the *Flavobacteriaceae* have also revealed their special ecological niche adaptive strategies in the oligotrophic ocean [10,11] or in the extremely cold environment [12].

*Flaviramulus ichthyoenteri* Th78^T^ is the type strain of the second established species in the genus *Flaviramulus* whose genomes have never been explored, and was isolated from the intestine of healthy flounder (*Paralichthys olivaceus*) in Qingdao, China, together with other four strains showed >99% 16S rRNA gene sequence similarity to Th78^T^ [13,14]. No such isolates were found in the rearing water or other parts of the flounder, indicating that these strains may prefer the intestinal environment. These *F. ichthyoenteri* isolates have been demonstrated to degrade various *N*-acylhomoserine lactones (AHLs); AHLs are widely used by Gram-negative bacteria as signal molecules in cell-to-cell communication (quorum sensing, QS) [14]. However, the underlying mechanism of this quorum quenching (QQ) property remains undiscovered.

Intestinal microorganisms play an important role in the nutrition and well-being of fish, and the nutrient-rich intestine of fish provides a favorable growth environment for numerous bacteria [15-20]. These bacteria can be categorized as either indigenous or transient species depending on their ability to colonize and adhere to the mucus layer [21]. To elucidate the ecological niche adaptations of *F. ichthyoenteri* Th78^T^ (including its QQ capability), its genome was sequenced and analyzed. Comparative genome studies with publicly available genome data of closely related flavobacterial strains were performed. Furthermore, the AHL degradation activity of candidate QQ enzymes was confirmed by recombinant expression in *Escherichia coli*. The results provide a first picture of the role of strain Th78^T^ in interacting with the intestinal environment and other intestinal bacteria, and reveal its potential to be used as a probiotic in aquaculture.

## Results and discussion

### Genome properties

The genome of *F. ichthyoenteri* Th78^T^ is 3,953,230 bp long (1 chromosome with no plasmid) with a G + C content of 32.06%. Of the 3,530 predicted genes, 3,451 are protein coding genes and 79 are RNAs. A total of six rRNA genes (two 5S rRNAs, two 16S rRNAs and two 23S rRNAs) and 40 tRNA genes were identified in the genome (Table 1). Among the predicted protein-coding genes, 925 (26.8%) can be assigned to COG categories and 1517 (43.9%), 3070 (88.9%), 844 (24.5%), and 3046 (88.3%) genes are found within the KEGG, NR, Swiss-Prot, and TrEMBL databases, respectively. The number of subsystems identified by RAST server is 331.

**Table 1:** **General features of** ***F. ichthyoenteri*** **Th78** ^**T**^ **,** ***G. saemankumensis*** **DSM 17032** ^**T**^ **and** ***Lacinutrix*** **sp. 5H-3-7-4** [22]

**Strains**	**Th78** ^**T**^	**DSM 17032** ^**T**^	**5H-3-7-4**
Size (bp)	3,953,230	3,089,149	3,296,168
G + C mol%	32.1%	34.3%	37.2%
Numbers of CDS	3,451	2,688	2,967
DNA coding percentage	90.22%	92.18%	91.64%
Numbers of rRNA	6	6	6
Numbers of tRNA	40	36	39
Major subsystem features predicted by RAST			
Cofactors, Vitamins, Prosthetic Groups, Pigments	171	172	153
Cell wall and capsule	129	77	105
Virulence, Disease and Defense	58	61	65
Membrane transport	89	63	53
RNA metabolism	128	125	121
Nucleosides and Nucleotides	78	80	74
Protein metabolism	172	173	167
DNA metabolism	91	83	70
Fatty Acids, Lipids, and Isoprenoids	58	54	72
Nitrogen metabolism	27	28	9
Respiration	46	44	32
Stress response	49	50	51
Amino acids and derivatives	272	259	252
Sulfur metabolism	48	31	6
Phosphorus metabolism	13	12	17
Carbohydrates	256	192	147

### General metabolism

For central carbohydrate metabolism, the genome of strain Th78^T^ encodes a full set of enzymes essential for carrying out Embden-Meyerhof-Parnas pathway which converts glucose into pyruvate and provide the precursors of metabolites for nucleotide and fatty acid biosynthesis. All the enzymes involved in the tricarboxylic acid cycle are also present. Pathways related to the utilization of various monosaccharides, disaccharides and aminosugars are predicted to be present in the genome of Th78^T^. Fructose and galactose can be converted to fructose-6-phosphate and glucose-1-phosphate respectively, and can be degraded through glycolysis. Sucrose can be converted to glucose-1-phosphate and fructose by a sucrose phosphorylase, and the ability to utilize sucrose has been confirmed experimentally in our previous study [13]. Consistent with the ability to use the amino sugar *N*-acetylglucosamine [13], two key enzymes, glucosamine-6-phosphate deaminase (NagB) and *N*-acetylglucosamine-6-phosphate deacetylase (NagA), are found in Th78^T^, as well as *N*-acetylglucosamine related transporters (NagX and NagP). For monosaccharide utilization, genes involved in ribose, L-fucose, mannose and xylose metabolism are present in the genome of Th78^T^. In addition, genes for D-galacturonate and D-glucuronate utilization are found (Additional file 1: Table S1). Most amino acids can be synthesized by strain Th78^T^, including animo acids essential for fish such as lysine, threonine, methionine, cysteine, leucine and histidine*.* All these features confer a metabolic versatility on strain Th78^T^ and allow it to participate in the conversion of material and energy in the fish intestine.

*F. ichthyoenteri* Th78^T^ is most likely to be a heterotrophic facultative member in the fish intestine, and it may have the potential for mixed acid fermentation in the anoxic intestine environment. According to KEGG pathway maps of Th78^T^, phosphoenolpyruvate (PEP) derived from glycolysis can be converted to oxaloacetate by a predicted phosphoenolpyruvate carboxylase and subsequently enter the TCA cycle to form succinate. The anaerobic dissimilation of pyruvate produced L- or D-lactate, acetate and acetyl-CoA; acetyl-CoA can be further fermented to butyrate-CoA. However, different from *Formosa agariphila* KMM 3901^T^ which has also been predicted to be capable of mixed acid fermentation [9], no pyruvate formate-lyase (PFL) is identified in Th78^T^, indicating that other enzymes are possibly used for pyruvate cleavage [23]. In addition, although Th78^T^ is unable to reduce nitrate to nitrite (due to the lack of nitrate reductase), a complete pathway of denitrification converting nitrite to nitrogen in the low oxygen environment can be found in its genome.

### Quorum quenching properties

QS is a mechanism of gene regulation in response to the concentration of specific signal molecules [24], and group behaviors such as biofilm formation and production of other virulence factors are coordinated by QS [25,26]. In Gram-negative bacteria including several aquaculture pathogens, such as *Edwardsiella tarda*, *Aeromonas* spp. and *Vibrio* spp., AHLs are the main signal molecules [27,28]. Thus QQ, namely the enzymatic interruption of QS, may be important to maintain homeostasis in gut ecosystem and inhibit the virulence of pathogenic microbes in the complicated intestinal environment.

**Figure 1:**
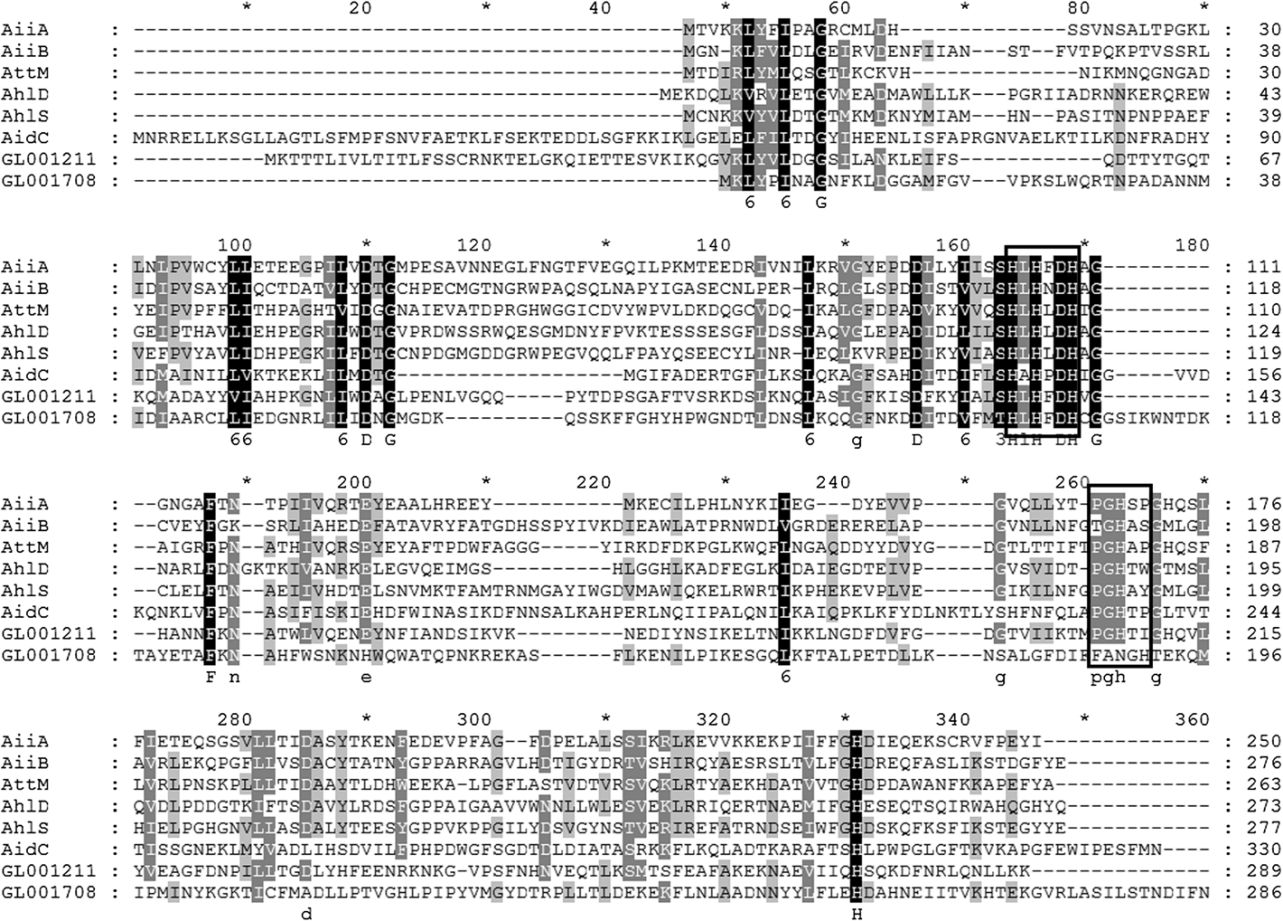
**Amino acid sequence comparison of six known AHL lactonases in metallo-β-lactamase superfamily and possible AHL lactones encoding by the genome of Th78** ^**T**^ **.** The two zinc-binding motifs are boxed with rectangles.

In our previous study [14], various QQ strains have been identified among the isolates from flounder. Among them, strain Th78^T^ was demonstrated to degrade various AHLs with different acyl chains including C6-homoserine lactone (HSL), 3-oxo-C6-HSL, C8-HSL, 3-oxo-C8-HSL, C10-HSL, 3-oxo-C10-HSL, C12-HSL, 3-oxo-C12-HSL, C14-HSL and 3-oxo-C14-HSL. According to the experimental results, the degrading activity in strain Th78^T^ was most likely attributed to AHL lactonases [14]. On that basis, bi-directional BLAST between all the predicted proteins of Th78^T^ and several reported AHL lactonases was performed. Two predicted β-lactamases (encoded by GL001211 and GL001708) show more than 25% identity with the reported AHL lactonases from metallo-β-lactamase superfamily (Table 2), and conserved zinc binding motif HXHXDH region has been identified in these two proteins (Figure 1). Three other proteins (encoded by GL001057, GL001387 and GL003427) are similar to AidH, an AHL lactonase from the α*/*β-hydrolase fold family. No hits are found when proteins from Th78^T^ were queried with QsdA [29] or AiiM [30].

**Table 2:** **Results of BLAST between all predicted proteins of Th78** ^**T**^ **and reported AHL lactonases**

Reported AHL lactonase*	**Protein in Th78** ^**T**^ **(Coding gene)**	**Identity (%)**	**Protein family**
AiiA (*Bacillus thuringiensis* KCTC 1507) [31]	GL001211	27.57	metallo-β-lactamase superfamily
	GL001708	25.15	
AiiB (*Agrobacterium tumefaciens* C58) [32]	GL001211	26.12	
	GL001708	25.34	
AttM (*Agrobacterium tumefaciens* C58) [33]	GL001211	27.89	
AhlD (*Arthrobacter* sp. IBN110) [34]	GL001211	26.55	
AhlS (*Solibacillus silvestris* StLB098) [35]	GL001211	25.46	
AidC (*Chryseobacterium* sp. StRB126) [36]	GL001708	28.94	
AidH (*Ochrobactrum* sp. T63) [37]	GL001057	23.20	α/β hydrolase fold family
	GL001387	34.00	
	GL003427	25.00	

*The amino acid sequences of reported AHL lactonases are obtained from Uniprot as follow: A3FJ64, A9CKY2, Q7D3U0, Q7X3T2, D2J2T6, I7HB71 and F8WSN1.

**Figure 2:**
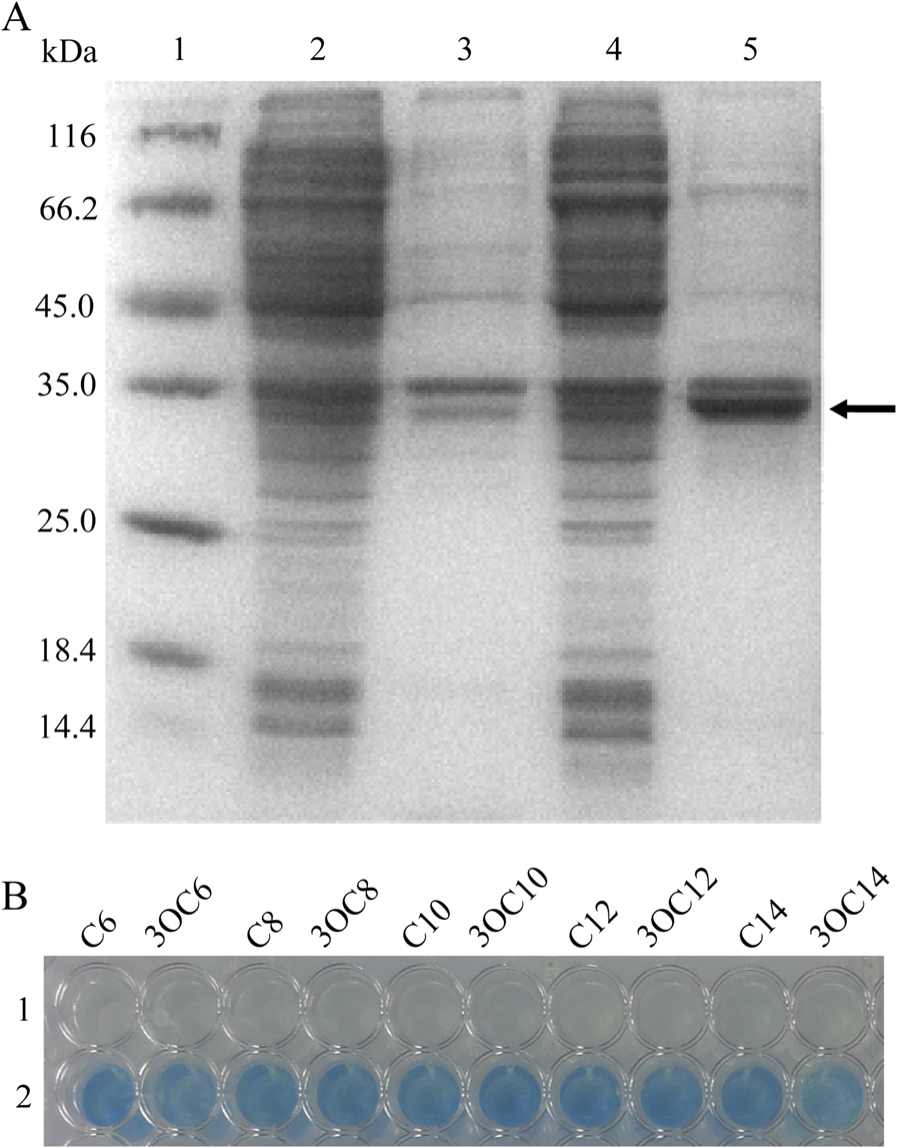
**Expression of FiaL and detection of its QQ activity. (A)**. SDS-PAGE analysis of overexpressed FiaL. Lane 1, protein molecular weight marker (Fermentas SM0431); Lane 2, supernatant of *E. coli*/pET 24a(+) after ultrasonication; Lane 3, resuspended cell pellet of *E. coli*/pET 24a(+) after ultrasonication; Lane 4, supernatant of *E. coli*/pET 24a(+)/GL001211 after ultrasonication; Lane 5, resuspended cell pellet of *E. coli*/pET 24a(+)/GL001211 after ultrasonication (the arrow indicates the position of FiaL). **(B)**. AHL-degrading activity of recombinant *E. coli* BL21(DE3). 1, supernatant of *E. coli*/pET 24a(+)/GL001211 after ultrasonication; 2, supernatant of *E. coli*/pET 24a(+) after ultrasonication.

Although the identities between predicted proteins of Th78^T^ and the reported AHL lactonases are relatively low, they still may be the active AHL lactonase considering the conserved zinc binding motif identified, so further experiments were performed to confirm the function of gene GL001211 and GL001708. The heterologous expression of gene GL001211 in *E. coli* led to a recombinant *E.coli* that is capable of degrading AHLs with different acyl-chain length and modifications (Figure 2). A protein with the expected mass (33 kDa) was produced (Figure 2). The name FiaL (*Flaviramulusichthyoenteri N*-acyl-homoserine lactonase) was proposed for this novel AHL-lactonase encoded by gene GL001211. However, the recombinant *E.coli* which expressed gene GL001708 did not show QQ activity.

FiaL homologs can be found in various marine bacteria of the family *Flavobacteriaceae*. FiaL shows the highest identity (70%) to a predicted protein in *Tenacibaculum ovolyticum* (WP_028890039), followed by *Polaribacter* sp. Hel_I_88 (WP_026776245, 63% identity) and *Aquimarina muelleri* (WP_027414219, 61% identity). However, the QQ activity of these homologous proteins remains to be confirmed experimentally.

### Digestive enzyme and vitamin production

Gut microorganisms might have a beneficial effect in the digestive process of fish as these bacterial isolates have been demonstrated to break down protein, chitin, cellulose, lipid, starch and phytate [38,39].

Th78^T^ was reported to be capable of hydrolyzing starch and sodium alginate in our previous study [13]. Starch may be hydrolyzed by a predicted 1, 4-α-glucan branching enzyme (GL001713) with an α-amylase domain, or other predicted glycoside hydrolase proteins. Four alginate lyases (GL000665, GL000671, GL000673 and GL000676) are identified, corresponding to the strong alginate hydrolysis ability in Th78^T^. Consistent with the lipase activity of strain Th78^T^, two putative lipases (GL002043 and GL003284) and two phospholipases (GL000298 and GL001418) are identified in its genome, and the breakdown of dietary lipids into fatty acids may promote the absorption of lipid in the intestine. Moreover, two xylanases (GL000440 and GL002553) in the genome of Th78^T^ indicate the ability to hydrolyze xylan which is a major component of hemicellulose. Although two genes encoding for chitinases (GL001289 and GL002608) are present in Th78^T^, no obvious chitinase ability was detected in our previous experiment.

Intestinal bacteria are also great sources of vitamins needed by the host. Strain Th78^T^ may be able to produce various B vitamins since homologs of most enzymes needed for the synthesis of biotin, riboflavin, pyridoxine, folate, nicotinate, thiamin and pantothenate are present in the genome of Th78^T^. This suggests that strain Th78^T^ may have a beneficial effect on the growth of fish and could potentially be useful as a probiotic in aquaculture.

### Utilization of substances in mucus

Mucus is a gel-like substance formed from glycoproteins (mucins) that contain negatively charged sugars (sialic acid or sulfosaccharide) [40]. It coats the surface of cells in digestive tract and has multiple roles in the intestinal environment [41].

Sialic acids comprise a family of nine-carbon amino sugars that are prevalent in mucus rich environments [42]. Several genes involved in sialic acid metabolism are found in the genome of Th78^T^, including a predicted *N*-acetyl neuraminate lyase (sialic acid lyase, NanA) and a tripartite ATP-independent periplasmic (TRAP) transporter for sialic acid. Catalyzed degradation of sialic acid by NanA yields pyruvate and *N*-acetyl-D-mannosamine (ManNAc), which makes sialic acid an attractive nutrient for microbes [43]. Sialidases are glycosylhydrolases that cleave the glycoketosidic linkages of Sia-O-acceptor substrates by an exohydrolytic reaction involving retention of configuration through a double-displacement mechanism [44]. Two genes encoding sialidases are identified, and this may allow Th78^T^ to scavenge host sialoglycoconjugates in the intestinal environment. Moreover, these sialidases in strain Th78^T^ may also be involved in regulation of host innate immunity, and play an important role in interspecies competition between sialidase-positive and sialidase-negative bacteria occupying the same niche [45,46].

Mucin in the intestine contained significant levels of sulfate covalently bound to the mucin oligosaccharide chains [47]. Sulfomucins may slow mucin degradation by mucin-degrading bacterial enzymes, and this role is thought to be particularly important in the colon. In the genome of strain Th78^T^, various kinds of sulfatases are predicted, including *N*-acetylgalactosamine-4-sulfatase, *N*-acetylgalactosamine-6-sulfatase and glucosamine-6-sulfatase, which could act on terminal or internal sulfated sugars of the mucin oligosaccharide chain and provide Th78^T^ an easier access to mucin-derived carbohydrates.

In addition to *N*-acetyl glucosamine and *N*-acetyl galactosamine, galactose and fucose are also contained in typical mucins [48]. Six β-galactosidase-encoding genes are identified in strain Th78^T^, which was confirmed by the experimental result in the API 20E and 20NE strips [13]. Strain Th78^T^ also has six genes coding for α-L-fucosidase (three of them are secretory), in agreement with the experimental result in API ZYM strip [13]. These α-L-fucosidases in Th78^T^ are capable of hydrolyzing various types of fucosidic linkages as well as synthetic substrates. α-L-fucosyl residues are frequently found at the terminal of many oligosaccharide chains in intestinal mucins, and fucosidases may play an important role in the intestinal ecosystem. All genes involved in the utilization of substances in mucus are summarized in Additional file 1: Table S2.

### General stress response

To successfully survive in the intestinal environment, *F. ichthyoenteri* Th78^T^ needs the ability to respond to a variety of stress conditions, and this may also enhance the potential of Th78^T^ to be used as a probiotic in the aquaculture [49]. The presence of bile acid is one of the main selective pressures in the intestine [50], and the mechanisms allowing intestinal bacteria to survive exposure to high bile acid concentrations include bile acid modifying enzymes and membrane transporters of these compounds [51,52]. One Na^+^ dependent transporter belonging to the sodium bile acid symporter family, which may contribute to bile acid resistance, was predicted by analysis of Th78^T^ genome. Intestinal bacteria are likely to be exposed to osmotic stresses because of diet fluctuation. Th78^T^ possesses two genes encoding for aquaporin Z which may increase the rate of water diffusion across cell membranes [53]. In addition, several enzymes which participate in the biosynthesis of the efficient osmoprotectant glycine betaine are identified, such as choline dehydrogenase (BetA) and choline sulfatase (BetC). Th78^T^ also encodes various enzymes dealing with oxidative stress including catalase, alkyl hydroperoxide reductase and superoxide dismutase, as well as various key regulators of the oxidative stress response. Molecular chaperones related to various stress responses in Th78^T^ include DnaK from the Hsp70 family, GroEL from the Hsp60 family, as well as ClpB and ClpC from the Hsp100 family. Cold shock proteins CspA and CspG are also present in Th78^T^.

### Gliding motility and type IX secretion system

Gliding motility is commonly found in the members of the phylum *Bacteroidetes* and is important in the competition for nutrients and colonization on surfaces [54]. Corresponding with the gliding motility of Th78^T^ [13], related genes were identified in its genome, including *gldABCDEFGHIJKLMN*, *sprA*, *sprE* and *sprT*. The amino acid sequences of Th78^T^ and *Flavobacterium johnsoniae* motility proteins [55,56] are similar, with identities ranging from 42% to 74%. Potential homologous proteins of SprB and RemA, which are mobile cell surface adhesins involved in gliding of *F. johnsoniae*, may also be encoded by the genome of Th78^T^ (33% and 30% identities, respectively). As a subset of gliding motility genes of Th78^T^, core genes for the type IX secretion system (*gldK*, *gldL*, *gldM*, *gldN*, *sprA*, *sprE*, and *sprT*) are present in the genome of Th78^T^ [57]. By using the TIGRFAM database, 39 genes are predicted to encode proteins with conserved C-terminal domains which are found in proteins known to be secreted by type IX secretion system [58], but the functions of most of these genes are unknown.

### Comparison with other *Flavobacteriaceae* genomes

*Gaetbulibacter saemankumensis* DSM 17032^T^ and *Lacinutrix* sp. 5H-3-7-4, which show relatively higher 16S rRNA gene sequence similarities (96.4% and 95.8%, respectively) and closer phylogenetic relationships with Th78^T^ among the genome-sequenced strains in *Flavobacteriaceae* (Additional file 2: Figure S1), were chosen for the comparative analysis with *F. ichthyoenteri* Th78^T^. *G. saemankumensis* DSM 17032^T^, the type species of *Gaetbulibacter*, was isolated from tidal flat sediment in Korea [59], while *Lacinutrix* sp. 5H-3-7-4 was reported as a polysaccharide-degrading strain from subseafloor sediment [22]. No in-depth genomic studies of these two strains have been performed yet. General features of these three genomes are compared in Table 1. The genome of Th78^T^ is larger than that of the other two sediment isolates, and the genome size of *Flavobacteriaceae* bacteria varies from approximately 2 ~ 6 Mb. According to the subsystems features predicted by RAST server, the extra genes of Th78^T^ mainly belong to subsystems such as carbohydrates, cell wall and capsule, membrane transport and sulfur metabolism.

**Figure 3:**
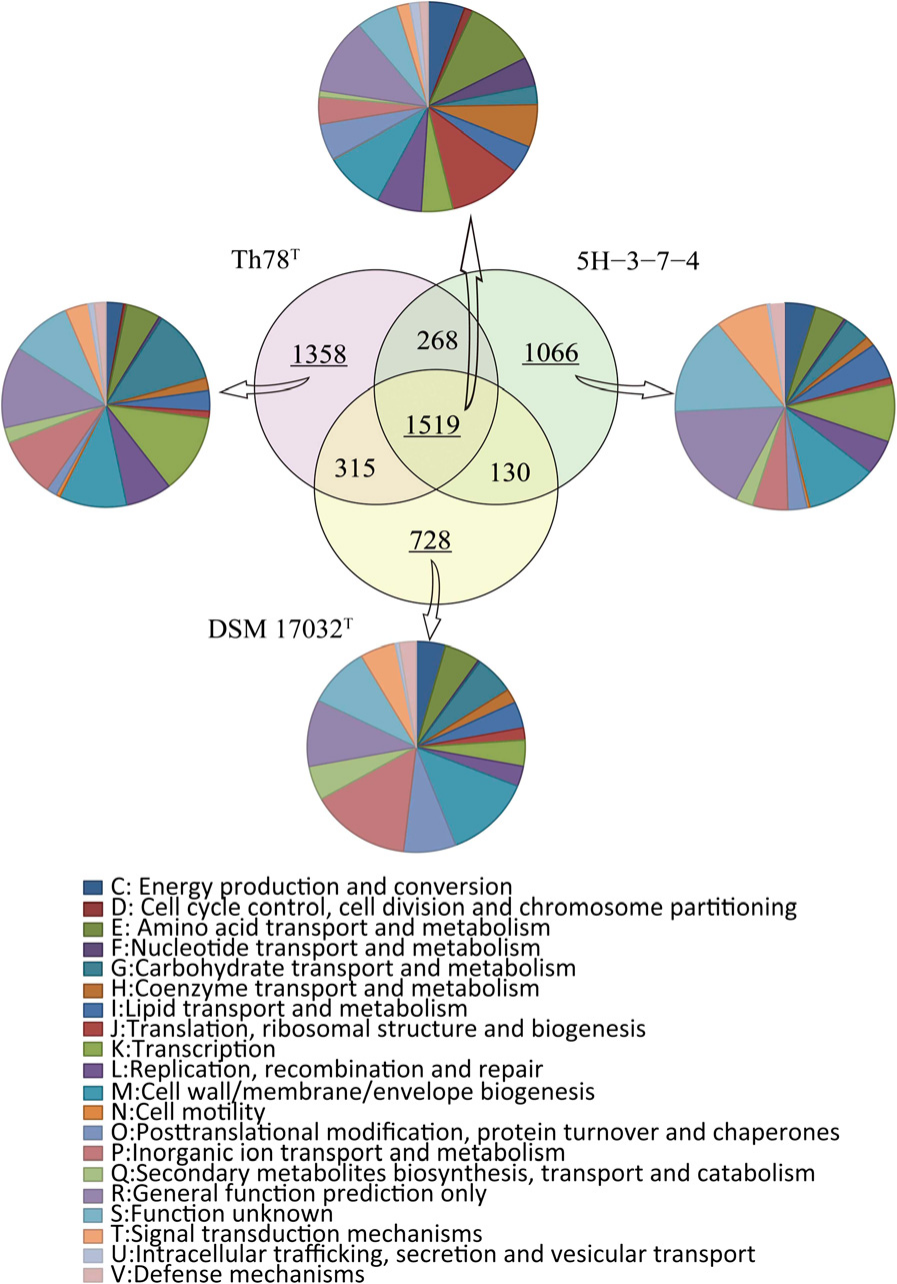
**Gene content comparison of Th78** ^**T**^ **,** ***G. saemankumensis*** **DSM 17032** ^**T**^ **and** ***Lacinutrix*** **sp. 5H-3-7-4.** The Venn diagram shows the orthologous and specific genes in each strain, and the pie charts show the relative abundance compared to all COG categories of the orthologous and specific genes in each strain.

Results of further comparative analysis based on all the predicted protein sequences of Th78^T^, DSM 17032^T^ and 5H-3-7-4 are shown in Figure 3. Although these three strains belong to different species in *Flavobacteriaceae*, they share a large number of orthologous genes (1519), accounting for 44.0%, 56.5% and 51.2% of all genes of Th78^T^, DSM 17032^T^ and 5H-3-7-4, respectively. Genes function in three COG categories, *i.e.* amino acid transport and metabolism (E), translation/ribosomal structure (J) and cell wall/membrane/envelope biogenesis (M), are highly conserved among three genomes. Corresponding with the larger genome of Th78^T^, it contains more specific genes (1358, 39.4%) than DSM 17032^T^ (728, 27.1%) and 5H-3-7-4 (1066, 35.9%). The largest proportion of specific genes with certain function in Th78^T^ are related to carbohydrate transport and metabolism (G) and cover genes encoding the following products: (i) α-L-fucosidases and fucose permeases, (ii) enzymes in aminosugar metabolism, (iii) enzymes in glucuronate metabolism, (iv) β-galactosidases, and (v) two complete TRAP-type C4-dicarboxylate transport systems which might be involved in sialic acid and glucuronate transportation (Additional file 1: Table S3). Th78^T^ is exposed to much more multiple and abundant carbohydrates in the intestine when compared to DSM 17032^T^ and 5H-3-7-4, thus evident distinctions can be found in carbohydrate utilization between Th78^T^ and those two marine sediment isolates. In addition, many specific genes of Th78^T^ are assigned to transcription (K), which encode for specific transcriptional regulators and AraC-type DNA-binding domain-containing proteins with unknown function. Generally there tends to be more transcriptional regulators in a larger genome [60], and this may help Th78^T^ to develop a sophisticated system to coordinate gene expression and thus adapt to the changing and competitive environment in fish intestine.

## Conclusions

Current studies on intestinal microbes mainly focus on some well-known and dominating genera in humans including *Bacteroides*, *Lactobacillus*, *Bifidobacterium*, *Fusobacterium*, *Enterobacteriaceae*, *etc*. Here we explore the potential features of a novel isolate from fish intestine, Th78^T^, by genome analysis. The AHL lactonase named FiaL was identified and expressed in *E.coli*, and this QQ property may confer competitive advantages on Th78^T^ in gut environment. Some signals used by aquaculture pathogens can be degraded by FiaL, such as C6-HSL (*Aeromonas hydrophila* and *Edwardsiella tarda*) [61,62], 3-oxo-C6-HSL (*Edwardsiella tarda* and *Vibrio salmonicida*) [63] and 3-oxo-C10-HSL (*Vibrio anguillarum*) [64]. Further analysis of Th78^T^ genome unveils that it may be able to produce various digestive enzymes and vitamins, which can be beneficial to the host. Th78^T^ also shows the ability to use some abundant nutrients in intestine, and this may be closely related to colonization and further influence the competitiveness. These results explained why Th78^T^ is able to successfully survive in the fish intestine, and further efforts may be needed to explore its potential to be used as a probiotics in aquaculture.

## Methods

### Growth conditions and DNA extraction

*F. ichthyoenteri* Th78^T^ was isolated from the intestine of cultured healthy flounder (*Paralichthys olivaceus*) in Qingdao, China and cultured on marine agar 2216 (MA; Becton Dickinson) at 28°C. Fish experiments were performed in compliance with Regulations for the Administration of Affairs Concerning Experimental Animals, and were approved by the Animal Ethics Committee of Shandong province. Genomic DNA of strain Th78^T^ was isolated by phenol-chloroform method [65].

### Genome sequencing and assembly

The genome of *F. ichthyoenteri* Th78^T^ was sequenced using Illumina Hiseq2000 sequencing platform with two libraries (500 bp and 6000 bp). A total of 907 Mb data was achieved constituting 229.4 fold coverage of the genome. The reads were assembled using SOAPdenovo assembler software [66], and a total of 17 contigs ranging from 246 bp to 1 394 011 bp (the N_50_ and N_90_ contig sizes were 878 943 bp and 267 282 bp, respectively) were obtained. By realigning the reads onto the contigs and using the paired-end information, these contigs were combined into five scaffolds. Intrascaffold gaps which were most likely comprised by repeats were closed using the pair-end extracted reads [66]. The largest scaffold was 3 944 211 bp, accounting for nearly 99.7% of the total length, and the other four scaffolds ranged from 637 bp to 6 595 bp.

### Nucleotide sequence accession numbers

This Whole Genome Shotgun project has been deposited at DDBJ/EMBL/GenBank under the accession AUYN00000000. The version described in this paper is version AUYN01000000.

### Genome annotation

Putative coding sequences (CDSs) were identified by Glimmer 3.0 [67]. RNAmmer [68] and tRNAscan [69] were used to predict rRNAs and tRNAs respectively, and sRNA was identified using Rfam database [70]. Functional annotation was performed by similarity analysis using the KEGG (Kyoto encyclopedia of genes and genomes; http://www.genome.jp/kegg/) [71], COG (http://www.ncbi.nlm.nih.gov/COG/) [72], SwissProt and TrEMBL (http://www.uniprot.org/), GO (Gene Ontology; http://www.geneontology.org/) [73], TIGRFAM [74] and NR (NCBI non-redundant database; http://www.ncbi.nlm.nih.gov/RefSeq/) [75]. The functional annotation and subsystem prediction were also performed using the RAST server [76]. The draft metabolic model was constructed by SEED Viewer version 2.0 (theSEED.org) [77].

### Expression of QQ enzymes and detection of QQ activity

The candidate QQ enzyme-encoding gene GL001211 was amplified by PCR with primer 1211-F (5'-CCG*GAATTC*ATGAAAACAACAACTC-3') and 1211-R (5'-CC*CTCGAG*TTTCTTTAATAAGTTTTG-3') (EcoRI and XholI sites are italics). The amplified DNA fragment was digested with EcoRI and XholI and was inserted into similarly digested pET-24a (+) (Novagen). Protein expression was performed with *E. coli* BL21 (DE3) (Novagen). To induce protein expression, IPTG (isopropyl-β-D-thiogalacto-pyranoside) was added to *E. coli* cultures grown to an optical density at 590 nm (OD_590_) of 0.4 ~ 0.5 at a final concentration of 0.1 mM. The induction was allowed to proceed for 12 h at 16°C. After incubation, cells were harvested by centrifugation and resuspended in PBS buffer (pH 6.8) for ultrasonication. The overexpressed protein was assessed by SDS-PAGE and *E. coli* BL21 (DE3) harboring an empty pET-24a (+) was used as negative control. To detect the QQ activity, supernatant obtained after induction and ultrasonication was mixed with certain concentration of AHLs (C6 to C14-HSL and 3-oxo-C6 to 3-oxo-C14-HSL) and the reaction was carried out at 28°C for 24 h. C4-HSL, C6-HSL, 3-oxo-C6-HSL and C8-HSL were purchased from Cayman Chemical Company (Ann Arbor, Michigan, USA); 3-oxo-C8-HSL, C10-HSL, 3-oxo-C10-HSL, C12-HSL, 3-oxo-C12-HSL, C14-HSL and 3-oxo-C14-HSL were purchased from Sigma-Aldrich (St. Louis, Missouri, USA). The biosensor *Agrobacterium tumefaciens* A136, which contains the AHL-responsive transcription factor TraR (pCF218) and the TraR-regulated *traI-lacZ* reporter (pCF372) [78], was used to detect the residual AHLs. Briefly, to make the A136 X-gal assay solution, an overnight broth culture of A136 was inoculated (1:100) into AT minimal glucose medium with X-gal at a final concentration of 250 μg mL^−1^. Ten μL reaction mixture and 190 μL A136 X-gal assay solution were added to 96-well plates, and degradation of AHLs was indicated by failing to express β-galactosidase activity in the biosensor strain (no blue color reaction) after incubating at 28°C for 24 h.

### Comparative genomics

The complete genome sequences of *G. saemankumensis* DSM 17032^T^ and *Lacinutrix* sp. 5H-3-7-4 were retrieved from NCBI. The general information about the genome of DSM 17032^T^ was obtained from IMG (http://img.jgi.doe.gov/). Proteins from Th78^T^ were compared with those of DSM 17032^T^ and 5H-3-7-4 using BLASTP with an E-value cutoff of 1e-5. Orthologous proteins are defined as reciprocal best hit proteins with a minimum 40% identity and 70% coverage, calculated by the BLAST algorithm [79]. Proteins without orthologs are considered to be strain-specific proteins. The COG function category was analyzed by searching all of the predicted proteins against the COG database on the basis of BLASTP.

## Additional files

Additional file 1: Table S1. Genes predicted to be involved in the general metabolism of *Flaviramulus ichthyoenteri* Th78^T^. **Table S2.** Genes predicted to be involved in the utilization of substances in mucus in *Flaviramulus ichthyoenteri* Th78^T^. **Table S3.** Specific genes predicted to be related to carbohydrate transport and metabolism (G) in *Flaviramulus ichthyoenteri* Th78^T^ when compared with *Gaetbulibacter saemankumensis* DSM 17032^T^ and *Lacinutrix* sp. 5H-3-7-4. Additional file 2: Figure S1. Neighbour-joining phylogenetic tree based on 16S rRNA gene sequences showing the phylogenetic position of strain Th78^T^ and closely related members of the family *Flavobacteriaceae*. Bootstrap percentages (>70%) based on 1000 replicates are shown at branch points. *Cryomorpha ignava* ACAM 647^T^ (GenBank accession no. NR027184) was used as an outgroup (not shown). Bar, 0.02 substitutions per nucleotide position. *indicates the strains whose genome sequence is available.
