# Supplementary material for: Genome analysis of Flaviramulus ichthyoenteri Th78T in the family Flavobacteriaceae: insights into its quorum quenching property and potential roles in fish intestine
Source: BMC Genomics. 2015 Feb 5;16(1):38. doi: 10.1186/s12864-015-1275-0 (PMC4324048; doi:10.1186/s12864-015-1275-0)
Supplement: Additional file 1: Table S1. — Genes predicted to be involved in the general metabolism of Flaviramulus ichthyoenteri Th78T. Table S2. Genes predicted to be involved in the utilization of substances in mucus in Flaviramulus ichthyoenteri Th78T. Table S3. Specific genes predicted to be related to carbohydrate transport and metabolism (G) in Flaviramulus ichthyoenteri Th78T when compared with Gaetbulibacter saemankumensis DSM 17032T and Lacinutrix sp. 5H-3-7-4. [file 12864_2015_1275_MOESM1_ESM.pdf]

Supplementary materials for

# **Genome analysis of *Flaviramulus ichthyoenteri* Th78$^T$ in the family *Flavobacteriaceae*: insights into its quorum quenching property and potential roles in fish intestine**

Yunhui Zhang, Jiwen Liu, Kaihao Tang, Min Yu, Tom Coenye and Xiao-Hua Zhang

**Table S1**. Genes predicted to be involved in the general metabolism of *Flaviramulus ichthyoenteri* Th78$^T$

| Gene description | Locus tag |
|---|---|
| **Embden-Meyerhof-Parnas pathway** | |
| Phosphoglucomutase (EC 5.4.2.2) | GL002623 |
| Glucose-6-phosphate isomerase (EC 5.3.1.9) | GL001412 |
| 6-Phosphofructokinase (EC 2.7.1.11) | GL000660 |
| | GL002949 |
| Fructose-bisphosphate aldolase (EC 4.1.2.13) | GL001002 |
| Triosephosphate isomerase (EC 5.3.1.1) | GL002715 |
| Glyceraldehyde 3-phosphate dehydrogenase (EC 1.2.1.12) | GL000345 |
| | GL002807 |
| | GL002950 |
| Phosphoglycerate kinase (EC 2.7.2.3) | GL001663 |
| Phosphoglycerate mutase (EC 5.4.2.1) | GL000966 |
| Enolase (EC 4.2.1.11) | GL002320 |
| Pyruvate kinase (EC 2.7.1.40) | GL001768 |
| **Tricarboxylic acid cycle** | |
| Pyruvate dehydrogenase (EC 1.2.4.1) | GL001108 |
| Dihydrolipoamide acetyltransferase (EC 2.3.1.12) | GL001107 |
| Dihydrolipoamide dehydrogenase (EC 1.8.1.4) | GL000252 |
| Citrate synthase (EC 2.3.3.1) | GL002322 |
| Aconitate hydratase (EC 4.2.1.3) | GL000525 |
| Isocitrate dehydrogenase (EC 1.1.1.42) | GL000579 |
| 2-Oxoglutarate dehydrogenase (EC 1.2.4.2) | GL001939 |
| Dihydrolipoamide succinyltransferase (EC 2.3.1.61) | GL000899 |
| Succinate-CoA ligase, alpha subunit (EC 6.2.1.5) | GL000069 |
| Succinate dehydrogenase iron-sulfur protein (EC 1.3.99.1) | GL001373 |
| Fumarate hydratase (EC 4.2.1.2) | GL003344 |
| Malate dehydrogenase (EC 1.1.1.37) | GL002799 |
| **Lactose and galactose metabolism** | |

| | |
|---|---|
| beta-Galactosidase (EC 3.2.1.23) | GL000453 |
| | GL000620 |
| | GL001193 |
| | GL001518 |
| | GL002468 |
| | GL002938 |
| Galactokinase (EC 2.7.1.6) | GL001039 |
| Galactose-1-phosphate uridylyltransferase (EC 2.7.7.12) | GL001038 |
| Fructose and mannose metabolism | |
| Xylose isomerase (EC 5.3.1.5) | GL002904 |
| Fructokinase (EC 2.7.1.4) | GL001086 |
| Mannose-6-phosphate isomerase (EC 5.3.1.8) | GL002706 |
| Phosphomannomutase (EC 5.4.2.8) | GL000183 |
| Mannose-1-phosphate guanylyltransferase (EC 2.7.7.22) | GL002329 |
| **Sucrose utilization** | |
| Sucrose phosphorylase (EC 2.4.1.7) | GL001085 |
| Predicted sucrose-specific TonB-dependent receptor | GL001089 |
| D-ribose utilization | |
| Ribokinase (EC 2.7.1.15) | GL001777 |
| | GL002093 |
| Ribose 5-phosphate isomerase (EC 5.3.1.6) | GL001692 |
| **Xylose utilization** | |
| Xylose isomerase (EC 5.3.1.5) | GL002904 |
| Xylulokinase (EC 2.7.1.17) | GL002905 |
| Xylanase | GL000400 |
| | GL002553 |
| Acetyl xylan esterase | GL002106 |
| | GL002551 |
| **D-galacturonate and D-glucuronate utilization** | |
| Altronate hydrolase (EC 4.2.1.7) | GL000464 |
| | GL002078 |
| Altronate dehydrogenases (EC 1.1.1.58) | GL002077 |
| Glucuronate isomerase (EC 5.3.1.12) | GL002073 |
| Mannonate dehydratase (EC 4.2.1.8) | GL002988 |
| 2-Dehydro-3-deoxygluconokinase (EC 2.7.1.45) | GL000661 |
| | GL002166 |
| 2-Dehydro-3-deoxyphosphogluconate aldolase (EC 4.1.2.14) | GL000659 |
| | GL002167 |
| Pectin degradation protein | GL000670 |
| | GL000970 |
| Gluconate 5-dehydrogenase (EC 1.1.1.69) | GL000716 |
| | GL002072 |
| 4-Deoxy-L-threo-5-hexosulose-uronate ketol-isomerase (EC 5.3.1.17) | GL000715 |
| | GL002071 |

| | |
|---|---|
| ***N*-acetylglucosamine utilization** | |
| N-acetylglucosamine-6-phosphate deacetylase (EC 3.5.1.25), NagA | GL002126 |
| Glucosamine-6-phosphate deaminase (EC 3.5.99.6), NagB | GL000434 |
| | GL001255 |
| | GL002128 |
| | GL002134 |
| Chitinase | GL001289 |
| | GL002608 |
| Hexosaminidase (EC 3.2.1.52) | GL000444 |
| | GL000477 |
| | GL000487 |
| | GL001161 |
| | GL001446 |
| N-acylglucosamine 2-epimerase (EC5.1.3.8) | GL002932 |
| N-acetylglucosamine related transporter, NagX | GL001261 |
| | GL003287 |
| N-acetyl glucosamine transporter, NagP | GL000436 |
| | GL002132 |

**Table S2**. Genes predicted to be involved in the utilization of substances in mucus in *Flaviramulus ichthyoenteri* Th78$^{T}$

| Gene description | Locus tag |
|---|---|
| **Sialic acids metabolism** | |
| *N*-acetyl neuraminate lyase (EC 4.1.3.3), nanA | GL002930 |
| Glucosamine-6-phosphate deaminase (EC 3.5.99.6), nagB | GL000434 |
| | GL002128 |
| | GL002134 |
| | GL001255 |
| Glucosamine-fructose-6-phosphate aminotransferase (EC 2.6.1.16), glmS | GL001437 |
| Sialidase (EC 3.2.1.18) | GL002933 |
| | GL002111 |
| Predicted sialic acid transporter | GL002939 |
| *N*-acylglucosamine 2-epimerase (EC 5.1.3.8) | GL002932 |
| UDP-*N*-acetylglucosamine 2-epimerase (EC 5.1.3.14) | GL000819 |
| | GL001632 |
| | GL001651 |
| *N*-acetylneuraminate synthase (EC 2.5.1.56), neuB | GL000822 |
| | GL001648 |
| Phosphoglucosamine mutase (EC 5.4.2.10) | GL000183 |
| Predicted *N*-acetylneuraminate transporter | GL000697 |

| | |
|---|---|
| *N*-Acetylneuraminate cytidylyltransferase (EC 2.7.7.43) | GL000823 |
| | GL002889 |
| *N*-acetylglucosamine-6-phosphate deacetylase (EC 3.5.1.25) | GL002126 |
| Sialic acid-specific 9-O-acetylesterase | GL002934 |
| **Degradation of glycosulfate esters** | |
| *N*-acetylgalactosamine 4-sulfatase (EC 3.1.6.12) | GL000611 |
| *N*-acetylgalactosamine-6-sulfatase (EC 3.1.6.4) | GL000621 |
| *N*-sulfoglucosamine sulfohydrolase (EC 3.10.1.1) | GL001525 |
| | GL002117 |
| Glucosamine-6-sulfatase (EC 3.1.6.14) | GL001523 |
| Choline-sulfatase | GL000705 |
| Uncharactitised sulfatases | (14 genes in total) |
| **Glycosidases** | |
| $\beta$-Galactosidase (EC 3.2.1.23) | GL000453 |
| | GL000620 |
| | GL001193 |
| | GL001518 |
| | GL002468 |
| | GL002938 |
| $\alpha$-L-Fucosidase (EC 3.2.1.51) | GL000458 |
| | GL000459 |
| | GL000461 |
| | GL000463 |
| | GL000469 |
| | GL000471 |

**Table S3**. Specific genes predicted to be related to carbohydrate transport and metabolism (G) in *Flaviramulus ichthyoenteri* Th78$^{T}$ when compared with *Gaetbulibacter saemankumensis* DSM 17032$^{T}$ and *Lacinutrix* sp. 5H-3-7-4.

| **Annotation by COG** | **Locus tag** |
|---|---|
| Glycerol uptake facilitator and related permeases | GL000389 |
| Beta-glucanase/ Beta-glucan synthetase | GL000421 |
| Fucose permease | GL000446 |
| Beta-glucanase/Beta-glucan synthetase | GL000453 |
| Alpha-L-fucosidase | GL000457 |
| Alpha-L-fucosidase | GL000458 |
| Alpha-L-fucosidase | GL000459 |
| Alpha-L-fucosidase | GL000461 |
| Alpha-L-fucosidase | GL000463 |
| Altronate dehydratase | GL000464 |
| Alpha-L-fucosidase | GL000469 |
| Alpha-L-fucosidase | GL000471 |

| | |
|---|---|
| N-acetyl-beta-hexosaminidase | GL000477 |
| N-acetyl-beta-hexosaminidase | GL000487 |
| Sugar phosphate isomerases/epimerases | GL000626 |
| Beta-glucosidase-related glycosidases | GL000656 |
| 6-phosphofructokinase | GL000660 |
| 6-phosphogluconate dehydrogenase | GL000675 |
| TRAP-type C4-dicarboxylate transport system, large permease component | GL000696 |
| TRAP-type C4-dicarboxylate transport system, small permease component | GL000697 |
| TRAP-type C4-dicarboxylate transport system, periplasmic component | GL000698 |
| 5-keto 4-deoxyuronate isomerase | GL000715 |
| N-acetyl-beta-hexosaminidase | GL001161 |
| Beta-galactosidase/beta-glucuronidase | GL001193 |
| Chitinase | GL001289 |
| Galactose mutarotase and related enzymes | GL001515 |
| Beta-glucosidase-related glycosidases | GL001527 |
| Sugar (pentulose and hexulose) kinases | GL001532 |
| 5-keto 4-deoxyuronate isomerase | GL002071 |
| Glucuronate isomerase | GL002073 |
| TRAP-type C4-dicarboxylate transport system, periplasmic component | GL002074 |
| TRAP-type C4-dicarboxylate transport system, small permease component | GL002075 |
| TRAP-type C4-dicarboxylate transport system, large permease component | GL002076 |
| Mannitol-1-phosphate/altronate dehydrogenases | GL002077 |
| Altronate dehydratase | GL002078 |
| Fructose/tagatose bisphosphate aldolase | GL002092 |
| Sugar kinases, ribokinase family | GL002093 |
| Fructose-1-phosphate kinase and related fructose-6-phosphate kinase (PfkB) | GL002094 |
| N-acetyl-beta-hexosaminidase | GL002100 |
| Sugar phosphate isomerases/epimerases | GL002123 |
| N-acetylglucosamine-6-phosphate deacetylase | GL002126 |
| 6-phosphogluconolactonase/Glucosamine-6-phosphate isomerase/deaminase | GL002128 |
| Fucose permease | GL002132 |
| 6-phosphogluconolactonase/Glucosamine-6-phosphate isomerase/deaminase | GL002134 |
| Beta-glucosidase-related glycosidases | GL002160 |
| Sugar kinases, ribokinase family | GL002166 |
| 2-keto-3-deoxy-6-phosphogluconate aldolase | GL002167 |
| Beta-galactosidase/beta-glucuronidase | GL002468 |
| Beta-galactosidase/beta-glucuronidase | GL002472 |
| Phosphomannose isomerase | GL002706 |
| Glucose/sorbosone dehydrogenases | GL002900 |
| Xylose isomerase | GL002904 |
| Sugar (pentulose and hexulose) kinases | GL002905 |
| Hydroxypyruvate isomerase | GL002909 |
| Sugar phosphate isomerases/epimerases | GL002919 |
| N-acyl-D-glucosamine 2-epimerase | GL002932 |

| | |
|---|---|
| Neuraminidase (sialidase) | GL002933 |
| N-acetyl-beta-hexosaminidase | GL002935 |
| Exo-beta-1,3-glucanase | GL002974 |
| Beta-galactosidase/beta-glucuronidase | GL002975 |
| Exo-beta-1,3-glucanase | GL002978 |
| Alpha-mannosidase | GL002982 |
| 3-carboxymuconate cyclase | GL002987 |
| D-mannonate dehydratase | GL002988 |
| Beta-galactosidase/beta-glucuronidase | GL002991 |
| Beta-fructosidases (levanase/invertase) | GL003137 |
| Predicted bile acid beta-glucosidase | GL003138 |
| Predicted bile acid beta-glucosidase | GL003140 |
