# Supplementary figures and images for: Genome analysis of Flaviramulus ichthyoenteri Th78T in the family Flavobacteriaceae: insights into its quorum quenching property and potential roles in fish intestine
Source: BMC Genomics. 2015 Feb 5;16(1):38. doi: 10.1186/s12864-015-1275-0 (PMC4324048; doi:10.1186/s12864-015-1275-0)

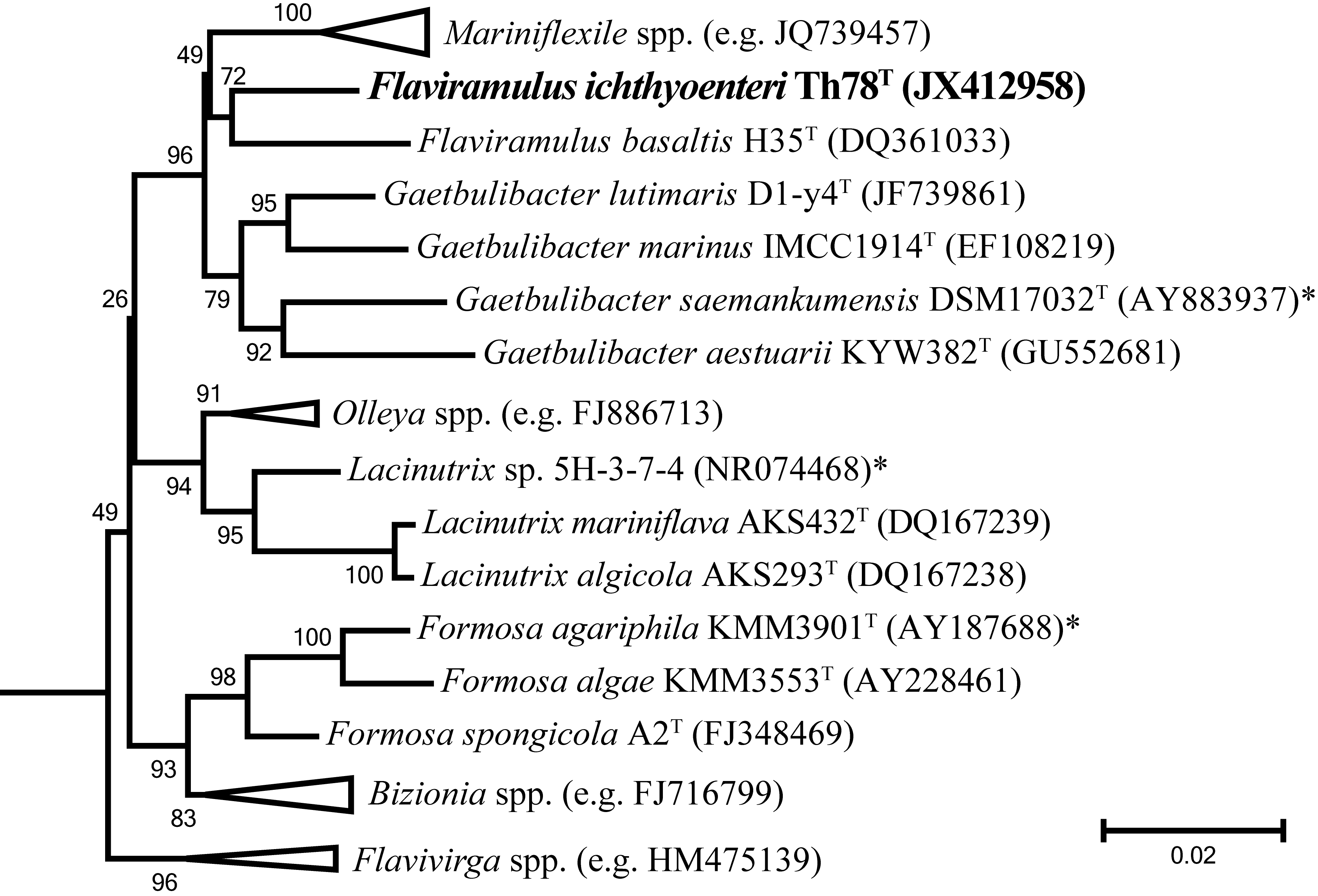

Supplement: Additional file 2: Figure S1. — Neighbour-joining phylogenetic tree based on 16S rRNA gene sequences showing the phylogenetic position of strain Th78T and closely related members of the family Flavobacteriaceae. Bootstrap percentages (>70%) based on 1000 replicates are shown at branch points. Cryomorpha ignava ACAM 647T (GenBank accession no. NR027184) was used as an outgroup (not shown). Bar, 0.02 substitutions per nucleotide position. *indicates the strains whose genome sequence is available. [file 12864_2015_1275_MOESM2_ESM.tiff]
